# Successful Treatment of Confirmed Severe Bupropion Cardiotoxicity With Veno-Arterial Extracorporeal Membrane Oxygenation Initiation Prior to Cardiac Arrest

**DOI:** 10.7759/cureus.53768

**Published:** 2024-02-07

**Authors:** Kyle D Pires, Joshua Bloom, Stephanie Golob, Barbara E Sahagún, Allison A Greco, Esha Chebolu, Jenny Yang, Peter Ting, Radu Postelnicu, Vanessa Soetanto, Leian Joseph, Sripal Bangalore, Sylvie F Hall, Rana Biary, Robert S Hoffman, David S Park, Carlos L Alviar, Rafael Harari, Silas W Smith, Mark K Su

**Affiliations:** 1 Division of Medical Toxicology, Ronald O. Perelman Department of Emergency Medicine, New York University Grossman School of Medicine, New York, USA; 2 New York City Poison Center, New York City Department of Health and Mental Hygiene, New York, USA; 3 Division of Medical Toxicology, Department of Emergency Medicine, The Warren Alpert Medical School of Brown University, Providence, USA; 4 The Leon H. Charney Division of Cardiology, New York University Grossman School of Medicine, New York, USA; 5 Ronald O. Perelman Department of Emergency Medicine, New York University Grossman School of Medicine, New York, USA; 6 Division of Pulmonary, Critical Care, & Sleep Medicine, New York University Grossman School of Medicine, New York, USA; 7 Department of Medicine, New York University Grossman School of Medicine, New York, USA; 8 Cardiac Intensive Care Unit and Department of Pharmacy, Bellevue Hospital Center, New York, USA

**Keywords:** ventricular dysrhythmia, cardiotoxicity, overdose, bupropion, va-ecmo

## Abstract

Bupropion is a substituted cathinone (β-keto amphetamine) norepinephrine/dopamine reuptake inhibitor andnoncompetitive nicotinic acetylcholine receptor antagonist that is frequently used to treat major depressive disorder. Bupropion overdose can cause neurotoxicity and cardiotoxicity, the latter of which is thought to be secondary to gap junction inhibition and ion channel blockade. We report a patient with a confirmed bupropion ingestion causing severe cardiotoxicity, for whom prophylactic veno-arterial extracorporeal membrane oxygenation (ECMO) was successfully implemented. The patient was placed on the ECMO circuit several hours before he experienced multiple episodes of hemodynamically unstable ventricular tachycardia, which were treated with multiple rounds of electrical defibrillation and terminated after administration of lidocaine. Despite a neurological examination notable for fixed and dilated pupils after ECMO cannulation, the patient completely recovered without neurological deficits. Multiple bupropion and hydroxybupropion concentrations were obtained and appear to correlate with electrocardiogram interval widening and toxicity.

## Introduction

Bupropion is a substituted cathinone (β-keto amphetamine) norepinephrine/dopamine reuptake inhibitor and noncompetitive nicotinic acetylcholine receptor antagonist that is used to treat major depressive disorder. It is also frequently prescribed for smoking cessation therapy, weight loss, attention deficit hyperactivity disorder, and compulsive eating disorders [1]. Data from United States poison centers indicate that bupropion is the antidepressant most frequently associated with fatalities [2].

Bupropion overdoses result in increased sympathetic tone, causing tachycardia, hypertension, agitation, and seizures. Based on animal data, cardiotoxicity is thought to be secondary to inhibition of both gap junctions and the human ether-a-go-go-related gene (hERG) channel delayed rectifier potassium current (IKr), causing QRS complex widening with systolic dysfunction and QT interval prolongation, respectively [3]. In notable contrast to other xenobiotics that cause QRS complex prolongation, bupropion does not directly inhibit sodium channels, even at significant doses [3]. Concordant with this, patients with reported bupropion overdoses present with QRS widening often unresponsive to hypertonic sodium bicarbonate, impaired inotropy, QT interval prolongation, cardiogenic shock, and malignant dysrhythmias [4-7].

Veno-arterial extracorporeal membrane oxygenation (VA-ECMO) is a form of mechanical hemodynamic support and oxygenation for a patient with low cardiac output and impaired perfusion. It is commonly used in patients with refractory cardiogenic shock and for patients with refractory cardiac arrest from ventricular tachycardia as extracorporeal cardiopulmonary resuscitation (ECPR) [8,9]. Data for extracorporeal membrane oxygenation (ECMO) as a bridge to recovery in poisoned patients are encouraging; poisoned patients have better outcomes than those cannulated for other indications, presumably due to the implied reversibility of their underlying pathophysiology [10]. Poison center data reveal thousands of bupropion exposures annually [2], of which only a small percentage develop severe cardiotoxicity. Prognostic criteria to predict which patients will develop severe toxicity following bupropion overdose have yet to be developed, and indications for VA-ECMO in patients with bupropion poisoning are not well-defined.

We report a patient with a confirmed bupropion ingestion causing severe cardiotoxicity for which VA-ECMO was successfully implemented. The patient was placed on the ECMO circuit prior to multiple episodes of ventricular dysrhythmias, which we believe was a critical contributing factor to his excellent outcome.

This work was accepted and presented in abstract form at the North American Congress of Clinical Toxicology in September 2023 (NACCT 2023, Montreal) and at the American Heart Association Scientific Sessions 2023 in November 2023 (AHA23, Philadelphia).

## Case presentation

**Figure 1:**
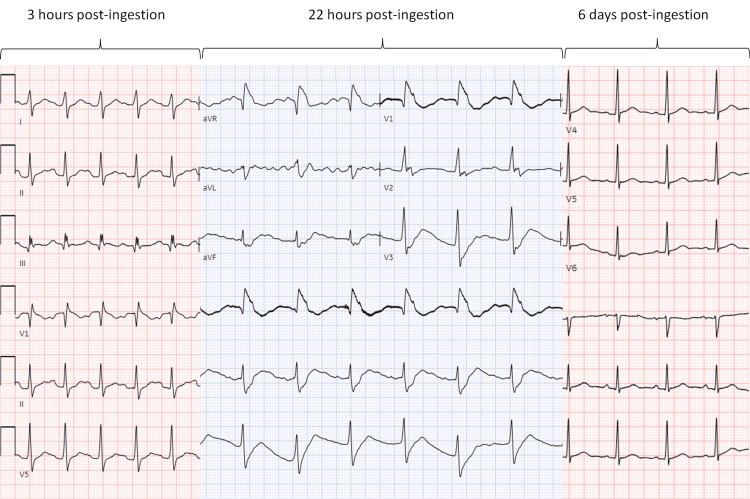
ECG evolution throughout the clinical course. The figure depicts the evolution of the ECG throughout care. Three hours post ingestion: sinus tachycardia with QRS of 110 ms and QTc of 544. Twenty-two hours post ingestion: QRS of 164 and QTc of 759, right axis shift, new right bundle branch block, and left anterior and posterior fascicular conduction disease. Six days post ingestion: normal sinus rhythm without prolonged intervals.

A 32-year-old transgender man (weight: 49.9 kg; height: 150 cm) with human immunodeficiency virus, major depressive disorder, and polysubstance use disorder presented to the emergency department after an intentional ingestion of bupropion three hours earlier. A self-terminating seizure occurred prior to arrival. Upon presentation, the initial vital signs were blood pressure (BP) of 125/82 mmHg, heart rate (HR) of 109 beats/minute, respiratory rate (RR) of 17 breaths/minute, and digital pulse oximetry of 99% (room air). A generalized tonic-clonic seizure occurred 20 minutes later, for which he received intravenous midazolam and was attached to a continuous electroencephalogram (EEG). An electrocardiogram (ECG) revealed a heart rate of 125 beats/minute, QRS complex duration of 110 ms, and QTc interval (Rautaharju’s correction) of 544 ms (Figure 1). These data in combination with another seizure approximately three hours after arrival prompted a recommendation for transfer to our tertiary referral center and VA-ECMO capable safety-net hospital.

**Figure 2:**
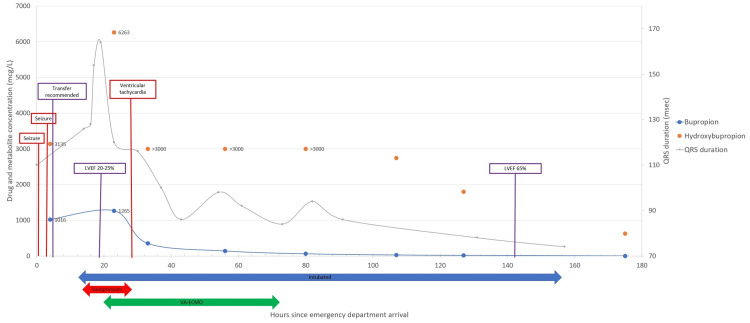
Clinical course, QRS duration, and bupropion/hydroxybupropion concentrations. The figure integrates key clinical aspects, QRS duration, and concentrations of bupropion and hydroxybupropion. VA-ECMO: veno-arterial extracorporeal membrane oxygenation; LVEF: left ventricular ejection fraction.

He was intubated for hypoxemia, airway protection, and elevated peak airway pressures necessitating bronchoscopy soon after arrival at our hospital, 13 hours from the initial presentation. The patient received 50 g of activated charcoal (AC) via an orogastric tube. One hour post-intubation, he developed shock requiring vasopressor support with norepinephrine. As seen in Figures 1, 2, serial ECGs showed rapidly progressively widening intervals, with a QRS complex duration that increased from 128 ms to 164 ms and a QTc duration that increased from 610 ms to greater than 750 ms over approximately two hours. These changes were associated with a clinical picture notable for increasing vasopressor requirements.

At 20 hours after initial presentation, with a QRS complex duration of 164 ms and QTc interval of greater than 750 ms, a bedside echocardiogram demonstrated a globally hypokinetic myocardium with a reduced left ventricular ejection fraction (LVEF) of 20-25%. This was a notable reduction in LVEF from approximately five hours earlier when a bedside echocardiogram estimated the LVEF to be 40-50%. Given the progression of cardiotoxicity as defined by ECG interval widening, increasing vasopressor requirements, and evidence of reduced LVEF, an emergent multidisciplinary shock team activation took place, including medical toxicology, medical critical care, critical care cardiology, interventional cardiology, advanced heart failure, and electrophysiology. The consensus was to pursue invasive hemodynamic assessment with a pulmonary artery catheter (PAC). The initial invasive hemodynamics revealed a cardiac index of 2.57 L/min/m2 (2.8-4.2 L/min/m2), right atrial pressure of 11 mm Hg (1-5 mm Hg), pulmonary artery pressure of 36/13 mm Hg (15/4-30/12 mm Hg), pulmonary artery pulsatility index of 2.1, and pulmonary capillary wedge pressure of 15 mm Hg (4-12 mm Hg). At that time, the patient’s vital signs were BP of 114/63 mm Hg, HR of 77 beats/minute, and O2 saturation of 100%, with laboratory serum parameters as seen in Table 1. A coronary angiogram was normal. These findings of normal hemodynamics and perfusion markers were compatible with a Society for Cardiovascular Angiography & Interventions (SCAI) SHOCK stage A, which implies that the patient is “at risk” of cardiogenic shock [11]. After a detailed discussion, the consensus was to proceed with prophylactic cannulation for VA-ECMO given the significant risk of ventricular dysrhythmias. The patient underwent cannulation 21 hours after presentation (24 hours post-ingestion, Figure 2).

**Table 1:** Laboratory studies prior to VA-ECMO cannulation. VA-ECMO: veno-arterial extracorporeal membrane oxygenation.

Laboratory serum study (units)	Concentration	Normal range
Lactate (mmol/L)	1.5	0.6 – 1.4
Creatinine (mg/dL)	0.8	0.5 – 0.9
Hemoglobin (g/dL)	11.9	12.0 – 16.0
Potassium (mmol/L)	3.6	3.5 – 5.1
Magnesium (mg/dL)	1.9	1.6 – 2.6

After cannulation, the patient’s pupils were fixed and dilated, for which an emergent noncontrast brain computed tomography scan was obtained, revealing no evidence of herniation, hemorrhage, or infarction. A second dose of 60 g AC was administered via orogastric tube 27 hours after presentation. Eight hours after ECMO initiation, he developed hemodynamically unstable monomorphic ventricular tachycardia (VT), which was treated with electrical cardioversion at 120J. He briefly remained in normal sinus rhythm, but VT recurred, and he was treated with a second electrical cardioversion at 200J. Normal sinus rhythm once again lasted for a few seconds before returning to VT. While preparing for the third electrical cardioversion, an intravenous lidocaine bolus of 100 mg followed by an infusion at 1 mg/min was administered; this was associated with a return to persistent sinus rhythm and a corresponding improvement in blood pressure, obviating repeat electrical cardioversion. Prior to the VT episode, the patient required norepinephrine at a rate of 3 mcg/min, which was increased to 10 mcg/min during the VT episode, and subsequently decreased to 1 mcg/min after the episode. The patient’s end organ perfusion was largely maintained with extracorporeal support during the hemodynamically unstable ventricular dysrhythmias, and there were no associated biomarker elevations indicative of end-organ damage. After this clinical nadir, he steadily improved with normalization of both the QRS and QT interval durations 37 hours after presentation. An echocardiogram performed 42 hours after the presentation revealed normalization of his LVEF. He was decannulated from the VA-ECMO circuit approximately 72 hours after ingestion. The patient was extubated three days later and was completely neurologically normal.

Serum bupropion (therapeutic range: 10-100 mcg/L) and hydroxybupropion (therapeutic range: 850-1500 mcg/L) concentrations were 1016 mcg/L and 3135 mcg/L at presentation, 1265 mcg/L and 6263 mcg/L three hours after ECMO initiation, 137 mcg/L and greater than 3000 mcg/L at ECMO decannulation, and 16 mcg/L and 1798 mcg/L at extubation (Figure 2).

## Discussion

National poison center data demonstrate that bupropion is the antidepressant reported to be associated with the largest number of severe outcomes in overdose [2]. Additionally, from 2012 to 2021, there has been a steady increase in the number of bupropion exposures as well as an increased proportion of severe outcomes [2]. While there are several case reports describing the use of ECMO in patients with bupropion overdose, co-ingestants have often confounded the clinical picture [12]. In all the previously reported cases in which cardiac arrest occurred, ECMO was initiated after the arrest as ECPR [5,7,13]. Only two of these cases confirmed the presence of bupropion with serum concentrations [5,7].

The lack of an effective bupropion antidote places patients at risk for cardiovascular collapse following overdose. We present a unique case of bupropion toxicity confirmed by serum concentrations, in which ECMO was initiated prior to cardiac arrest thereby providing hemodynamic support and maintaining perfusion during malignant dysrhythmias, which contributed to an excellent outcome. Similarities to other case reports include initial seizures, early severe and rapidly progressive cardiotoxicity, and apparent brain death [5-7,14].

The potential role of lidocaine in bupropion-associated wide complex dysrhythmias is useful to explore. For this patient, we hypothesize that monomorphic ventricular tachycardia was likely due to reentry in the setting of marked conduction defects due to gap junction inhibition. We posit that lidocaine, as a Vaughan-Williams class 1b antidysrhythmic and sodium channel blocker, functioned to terminate the re-entrant loop without further prolonging the QT interval duration and increasing the risk of torsade de pointes (as a class 3 anti-arrhythmic might). Lidocaine is therefore a reasonable choice for termination of monomorphic VT in patients with bupropion cardiotoxicity and a prolonged QT interval.

Analysis of the serum bupropion concentrations over time revealed first-order toxicokinetics with an apparent half-life of 17.6 hours (starting from the bupropion concentration obtained at 32 hours since presentation, see timeline in Figure 2). Hydroxybupropion concentrations were unable to be formally interpreted, as sufficient dilutions were not completed for several samples. It should be noted that the patient appeared to have continued gastrointestinal absorption of bupropion over 24 hours after ingestion. This underscores the importance of gastrointestinal decontamination in patients with bupropion overdose, especially given the lack of available antidotal therapy. Given its ability to bind bupropion in vitro [15], AC should be administered to those patients who can safely tolerate oral intake and to all patients with a protected airway. Other forms of gastrointestinal decontamination, such as gastric lavage and whole bowel irrigation, should be discussed with a consulting toxicologist. Interestingly, this patient’s peak QRS complex and QTc interval widening closely correlated with peak bupropion and hydroxybupropion concentrations. Consistent with other published data, cardiotoxicity appears to be better correlated with bupropion concentrations as opposed to hydroxybupropion concentrations [16]. This patient was able to undergo VA-ECMO decannulation when bupropion concentrations were in the therapeutic range, while hydroxybupropion concentrations remained elevated for several days.

It is difficult to prognosticate bupropion overdose severity. For this patient, our recommendation to transfer to an ECMO center was based on recurrent seizures and QRS/QTc prolongation. The decision to proceed with VA-ECMO cannulation by our multidisciplinary team was informed by widening ECG intervals and echocardiographic evidence of worsening LVEF despite normal hemodynamic status and early cardiogenic shock state (SCAI SHOCK stage A) [11].

## Conclusions

The use of VA-ECMO can be lifesaving in patients with severe bupropion poisoning. While specific criteria to initiate VA-ECMO are currently unknown, progressive ECG interval widening, reduction in ejection fraction on echocardiography, or cardiogenic shock should prompt shock team consultation and VA-ECMO consideration prior to cardiovascular collapse. We would like to underscore the importance of a multi-disciplinary shock team discussion as traditional SCAI SHOCK staging for this patient would not suggest the use of mechanical circulatory support. However, given the concerns raised with respect to gap junction inhibition, lack of antidotal therapeutic options, and rapid progression of conduction abnormalities, a consensus was reached that the benefit of prophylactic VA-ECMO likely outweighed the risks of this therapy. While it is impossible to know what would have occurred if a watchful waiting strategy were to have been employed, it stands to reason that ECPR initiation at the time of the malignant dysrhythmia in this patient could have resulted in a delay to hemodynamic stabilization and thus an increased risk of morbidity and mortality. With regards to antidysrhythmic therapy, this individual case suggests that lidocaine is a reasonable choice for the termination of monomorphic VT in patients with bupropion cardiotoxicity and a prolonged QT interval; however, this would benefit from further investigation. While this patient demonstrated QRS complex and QTc prolongation that appeared to be most severe when bupropion concentrations were at their peak, additional research is required to understand the potential correlation between serum bupropion concentrations and ECG changes or clinical deterioration.
